# Genomic Characterization of *Streptococcus suis* Serotype 24 Clonal Complex 221/234 From Human Patients

**DOI:** 10.3389/fmicb.2021.812436

**Published:** 2021-12-23

**Authors:** Anusak Kerdsin, Rujirat Hatrongjit, Thidathip Wongsurawat, Piroon Jenjaroenpun, Peechanika Chopjitt, Parichart Boueroy, Nahuel Fittipaldi, Han Zheng, Marcelo Gottschalk

**Affiliations:** ^1^Faculty of Public Health, Kasetsart University Chalermphrakiat Sakon Nakhon Province Campus, Sakon Nakhon, Thailand; ^2^Department of General Sciences, Faculty of Science and Engineering, Kasetsart University Chalermphrakiat Sakon Nakhon Province Campus, Sakon Nakhon, Thailand; ^3^Division of Bioinformatics and Data Management for Research, Department of Research and Development, Faculty of Medicine Siriraj Hospital, Mahidol University, Bangkok, Thailand; ^4^Research Group on Infectious Diseases in Production Animals (GREMIP), Faculty of Veterinary Medicine, University of Montreal, Quebec, QC, Canada; ^5^State Key Laboratory of Infectious Disease Prevention and Control, Collaborative Innovation Center for Diagnosis and Treatment of Infectious Diseases, Chinese Center for Disease Control and Prevention, National Institute for Communicable Disease Control and Prevention, Beijing, China

**Keywords:** sequence type, serotype, genome, *Streptococcus suis*, antimicrobial-resistant gene

## Abstract

*Streptococcus suis* is a zoonotic pathogen that causes invasive infections in humans and pigs. Although *S. suis* serotype 2 is prevalent among patient and swine infections, other serotypes are occasionally detected in humans. Of these, serotype 24 clonal complex (CC) 221/234 are recognized as emerging clones of human infection. Genomic exploration of three *S. suis* serotype 24 CC221/234 strains revealed antimicrobial resistance genes, pathotyping, virulence-associated gene (VAG) profiles, minimum core genome (MCG) typing, and comparison of the genomes. Based on these analyzes, all three serotype 24 strains were MCG7-3 and should be classified in the intermediate/weakly virulent (I/WV) group. All selected serotype 24 strains were susceptible to several antibiotics including β-lactam, fluoroquinolone, and chloramphenicol. Resistance to tetracycline, macrolide, and clindamycin was observed and attributed to the genes *tet(O)* and *erm(B)*. Genomic comparison revealed the strains S12X, LSS66, LS0L, LS0E, 92–4,172, and IMT40201 that had phylogenetic affinity with serotype 24 CC221/234. Analysis of 80 virulence-associated genes (VAG) showed that all three serotype 24 strains lacked 24 genes consisting of adhesin P, *epf, hyl, ihk, irr, mrp, nadR, neuB, NisK/R, ofs*, permease *(SSU0835), rgg, revS, salK/R, sao, sly, spyM3_0908, srtBCD, srtF, srtG, SSU05_0473, virA, virB4*, and *virD4.* Eleven specific sequences were identified in the 3 serotype 24 genomes that differed from the genomes of the representative strains of epidemic (E; SC84), highly virulent (HV; P1/7), I/WV (89–1,591), and avirulent (T15 and 05HAS68).

## Introduction

*Streptococcus suis*, an important swine pathogen, also causes invasive infections in humans who have been in close contact with infected pigs or contaminated pork-derived products (Goyette-Desjardins et al., 2014). The number of reported human cases, especially in Southeast Asian countries, has substantially increased in the past few years (Goyette-Desjardins et al., 2014; Segura, 2020). Among the 29 described serotypes of *S. suis*, serotype 2 is the most common cause of human infections (Goyette-Desjardins et al., 2014; Okura et al., 2016). However, human cases have also been reported due to the rare serotypes 4, 5, 7, 9, 14, 16, 21, 24, and 31 (Nghia et al., 2008; Callejo et al., 2014; Goyette-Desjardins et al., 2014; Hatrongjit et al., 2015; Kerdsin et al., 2017; Liang et al., 2021).

*S. suis* strains affecting humans include sequence types (STs) 1, 3, 7, 9, 11, 20, 25, 28, 101, 102, 103, 104, 105, 107, 126, 134, 144, 146, 233, 298, 337, 379, 380, 381, 382, 391, 392, 393, 395, 512, 513, 514, 515, and 516, based on multilocus sequence typing (MLST) (Goyette-Desjardins et al., 2014; Kerdsin et al., 2018). However, most human clinical STs are grouped into limited clonal complexes (CCs) consisting of CC1, CC16, CC94, CC20, CC25, CC28, CC104, CC221/234, and CC233/379 (Okura et al., 2016; Hatrongjit et al., 2020). CC1 has been found worldwide, including in Europe, Asia, Australia, and South America, while CC20 (ST20) has been described as important in the Netherlands (Hatrongjit et al., 2020). Furthermore, ST7 (CC1), responsible for the 1998 and 2005 epidemics, was mostly present in China, while CC16 and CC94 were predominant in Europe, although human cases were reported in Thailand (Hatrongjit et al., 2020). CC25 or CC28 were reported in North America and were also recovered in Thailand, Korea, Japan, and Australia (Hatrongjit et al., 2020). Finally, CC104, CC221/234, and CC233/379 were endemic to Thailand (Hatrongjit et al., 2020).

In previous study, we reported three human cases of *S. suis* serotype 24 that belonged to the clonal complex (CC) 221/234 (Kerdsin et al., 2018). Of these three cases, two and one belonged to ST221 and ST234, respectively. *S. suis* serotype 24 CC221/234 has also been documented in Thailand since 2011 (Kerdsin et al., 2011). To date, seven human cases infected by *S. suis* CC221/234 strains have been reported; five of them were serotype 24 and the rest belonged to serotypes 5 and 31 (Kerdsin et al., 2011, 2016, 2018; Hatrongjit et al., 2015). Among seven *S. suis* CC221/234 cases, four were documented in northern Thailand, whereas two cases and one case were reported in central and eastern Thailand, respectively (Kerdsin et al., 2011, 2016, 2018; Hatrongjit et al., 2015). CC221/234 is an emergent clone that has caused human infections in Thailand (Hatrongjit et al., 2020).

Whole-genome sequencing (WGS) approaches have increasingly been used to investigate *S. suis* strains. Among 29 serotypes, WGS has been applied to the serotype 2 strains worldwide for, as examples, characterization of outbreaks, evaluation of *S. suis* reinfection, determining the population structure of *S. suis* strains, and identifying pathotypes or virulence traits (Estrada et al., 2019; Hatrongjit et al., 2020). Several studies have also been conducted on serotypes other than serotype 2 including serotypes 3–9, 14, 16, 19, 20, 21, 22, 26, and unencapsulated strains, although most of them were of swine origin (Hu et al., 2011; Wang et al., 2013a,b, 2014; Baig et al., 2015; Yoshida et al., 2017; Zheng et al., 2018; Niemann et al., 2019; Stevens et al., 2019; Bunk et al., 2021; Liang et al., 2021; Nicholson et al., 2021). To date, there has been no WGS performed on *S. suis* serotype 24 strains recovered from humans. Herein, we describe the genomic analysis of three *S. suis* serotype 24 CC221/234 strains recovered from human patients (Kerdsin et al., 2018). This study could provide insight into genomic characteristics, putative virulence genes, genetic relationships, and the prediction of pathogenic capacity of this serotype.

## Materials and Methods

### Bacterial Strains and Antimicrobial Susceptibility

Three *S. suis* serotype 24 strains, belonging to CC221/234, were recovered from blood samples of individual sepsis cases and consisted of two ST221 (ID33329 and ID39565) and one ST234 (ID32098). ID39565 was recovered in 2012 from Central Thailand, whereas the ID33329 and ID32098 strains were isolated in 2010 in Northern Thailand (Kerdsin et al., 2018).

The broth microdilution technique was used according to the standards defined in the M100 (31st edition) Clinical and Laboratory Standard Institute (CLSI) guidelines to determine the minimum inhibitory concentrations (MICs) of penicillin (≤0.12 μg/ml = susceptible; 0.25–2 μg/ml = intermediate; ≥ 4 μg/ml = resistance) (Clinical and Laboratory Standards Institute [CLSI], 2021). Susceptibility to other antimicrobials, such as ceftriaxone cefepime, azithromycin, erythromycin, tetracycline, clindamycin, levofloxacin, and chloramphenicol, was determined using the disk diffusion technique following the 2021 CLSI-M100 guidelines (Clinical and Laboratory Standards Institute [CLSI], 2021). Since there are currently no breakpoints recommended for *S. suis*, those for the viridans group streptococci were used, as defined in the guidelines (Clinical and Laboratory Standards Institute [CLSI], 2021). The *Streptococcus pneumoniae* ATCC 49619 strain was used for quality control purposes.

### Whole-Genome Sequencing

Bacterial genomic DNA samples extracted using ZymoBIOMICS DNA Kits (Zymo Research, CA, United States) were sequenced using the Oxford Nanopore Technologies (ONT) and Illumina platforms. Library preparation for ONT sequencing followed the rapid barcoding DNA sequencing protocol with the SQK-RBK004 kit without DNA size selection (preserve the plasmid DNA) and the libraries were sequenced using a single R9.4.1/FLO-MIN106 flow cell on a MinION Mk1B sequencer. We base called and demultiplexed the raw data using Guppy v3.4.5 (ONT) specifying the high-accuracy model (-c dna_r9.4.1_450bps_hac.cfg). The ONT adapters were trimmed using Porechop v0.2.4.^1^ Quality control of ONT reads was undertaken using Nanoplot v1.28.1.^2^ For the Illumina platform, the sequencing library was generated using a NEBNext Ultra II DNA Library Prep Kit for Illumina (New England Biolabs, United Kingdom), following the manufacturer’s recommendations. The genomic DNA was randomly fragmented to a size of 350 bp and the fragments were A-tailed and ligated with the adapter. Libraries were sequenced using the Illumina HiSeq platform with the 150 paired-end sequencing strategy. We applied Fastp v0.19.5 (14) with default parameters for the quality filtering of Illumina reads. Adapters were trimmed using Skewer v0.2.2 (Jiang et al., 2014). The quality checking of Illumina reads was performed using FastQC v0.11.8.^3^ Hybrid assemblies with the ONT and Illumina data were performed using Unicycler v0.4.8 (Wick et al., 2017) and the genome sequences were checked for quality using QUAST v5.0.2 (Gurevich et al., 2013). Genome sequences were submitted to the NCBI Prokaryotic Genome Annotation Pipeline (PGAP v4.12) for annotation. The default parameters were used for all software unless otherwise specified.

### Bioinformatics Analysis

Antimicrobial resistance genes were detected using ResFinder 4.1 (Bortolaia et al., 2020). Plasmid replicons were analyzed using PlasmidFinder 2.1 and PLACNETw (Carattoli et al., 2014; Vielva et al., 2017). Sequence type (ST) was confirmed using the PubMLST database.^4^ Minimum core genome (MCG) sequence typing was done according to a procedure described elsewhere (Chen et al., 2013a). The completed genome of strain ID39565 was used as the reference genome for comparative genomic analysis using BRIG (BLAST Ring Image Generator) v0.95 (Alikhan et al., 2011), and the results were used to draw a circular graphic of the genome comparison. The core- and pan-genome analyses of the *S. suis* strain ID39565, ID32098, and ID33329 were performed using Roary v3.13.0 (Page et al., 2015). The core and accessory genes among the three genomes were counted and visualized using VennDiagram v1.6.2 (Chen and Boutros, 2011).

Eighty virulence-associated genes (VAG) described in *S. suis* were used to determine their presence in the serotype 24 strains using MyDbFinder 2.0, Center for Genomic Epidemiology (Supplementary Table 1; Fittipaldi et al., 2012; Zheng et al., 2014a,^2018^; Estrada et al., 2021). Out of 80 VAG, the presence or absence of 22 VAG described in a previous study was analyzed using unweighted average linkage (UPGMA) with the DendroUPGMA program via http://genomes.urv.cat/UPGMA/ (Garcia-Vallvé et al., 1999; Dong et al., 2015). Mobile genetic elements, restriction-modification (RM) system, and the clustered regularly interspaced short palindromic repeats (CRISPR)-Cas system were analyzed using Mobile Element Finder (Johansson et al., 2021), Restriction-Modification Finder (Roer et al., 2016) via Center for Genomic Epidemiology,^5^ and CRISPRCasFinder^6^; Couvin et al., 2018), respectively.

To search for the genetically closest relatives to the three serotype 24 strains, a modular single genome analysis was conducted following the core genome multilocus sequence typing (cgMLST) approach by BacWGSTdb 2.0 (Feng et al., 2021). The genetically closest relatives were chosen for 5–10 strains based on small numbers of allelic differences with selection thresholds of 100–500, depending on the strains under current study. The phylogenetics of the serotype 24 strains and the closest relatives selected from BacWGSTdb were conducted using a reference genome-based single-nucleotide polymorphism (SNP) strategy with CSI phylogeny (Kaas et al., 2014). The phylogenetic tree was visualized using the iTOL V4 software (Letunic and Bork, 2019). *S. suis* serotype 2 S735, a type strain (accession no. CP003736), was used as the reference sequences for SNP analysis.

In addition, genome comparisons were undertaken between the three genomes of the serotype 24 strains to serotype 2 genomes of epidemic (E) strain SC84 (FM252031), highly virulent (HV) strain P1/7 (CP003736), intermediate/weakly virulent (I/WV) strain 89–1,591 (GCA_000440595), and the avirulent strains T15 (CP006246) and 05HAS68 (CP002007) (Jiang et al., 2009; Zheng et al., 2014b; Yao et al., 2015), using the Mauve software and following the previous instructions (Darling et al., 2010). BLASTN was used to search for the sequence homology of unique genes or coding sequences identified in the serotype 24 strains.^7^

### Accession Number

The genome sequences of the three *S. suis* serotype 24 strains were deposited in the NCBI GenBank under Bioproject accession number PRJNA691075 with Genbank accession numbers of CP068708, CP076517, and CP082778 for the strain no. ID33329, ID39565 and ID32098, respectively.

## Results and Discussion

### General Genomic Information

The completed genomes of the three *S. suis* serotype 24-CC221/234 were 2,138,155 bp, 2,169,193 bp, and 2,137,421 bp for strains ID33329 (ST221), ID39565 (ST221), and ID32098 (ST234), respectively. Strain no. ID33329 contained 2,047 genes, 1,975 coding sequences (CDS), 4 of each gene 5S rRNA, 16S rRNA, and 23s rRNA, 56 tRNA genes, and 4 ncRNA genes. The ID39565 had 2,100 genes, 2,028 CDS, 4 of each gene 5S, 16S, and 23S rRNA genes, 56 tRNA genes, and 4 ncRNA genes. ID32098 contained 2,051 genes, 1,979 CDS, 4 of each gene 5S, 16S, and 23S rRNA genes, 56 tRNA genes, and 4 ncRNA genes.

**FIGURE 1:**
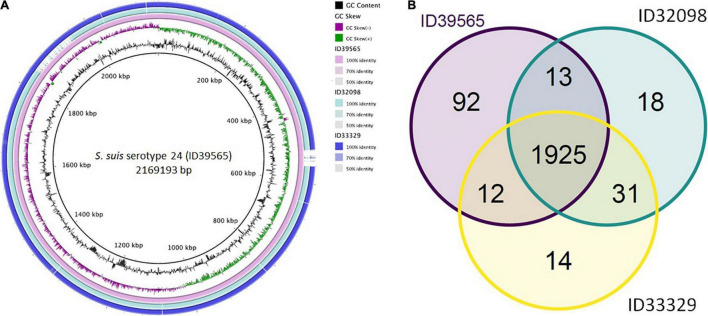
**(A)** Comparative genomic circular map of *Streptococcus suis* strain ID39565 constructed using BRIG v0.95. The features are as follows (ring from center to outside): ring 1 is genome size; ring 2 is GC content; ring 3 is GC skew; rings 4 to 6 are comparative genomic maps of *S. suis* strains ID39565, ID32098, and ID33329, respectively, with ID39565 genome as the reference. Blank spaces in the ring represent matches with less than 50% identity to the reference genome (ID39565). **(B)** Venn diagram depicting unique and shared genes between the genomes of the *S*. *suis* serotype 24 strains ID39565 (left), ID32098 (right), and ID33329 (bottom). Each strain is represented by one color, and the number of genes is displayed in the same color. Numbers in intersectional regions indicate genes shared by two or three strains.

As shown in Figure 1, comparative genomic analysis and a Venn diagram of three *S. suis* serotype 24-CC221/234 strains revealed 92, 18, and 14 unique genes present only in strains ID39565, ID32098, and ID33329, respectively. In addition, 1,925 genes were commonly found in these three strains, whereas 13 genes were present in strains ID39565 and ID32098, and 12 genes were found in strains ID39565 and ID33329, respectively. There were 31 genes present in strains ID32098 and ID33329.

### Antimicrobial Resistance

As recently reported with other strains (Bamphensin et al., 2021), the MIC values on penicillin revealed intermediate resistance for strains ID33329-ST221 (MIC of 0.75 μg/ml) and ID32098-ST234 (MIC of 0.38 μg/ml). These two strains had numerous substitutions in PBP2B and PBP2X, as described elsewhere (Bamphensin et al., 2021). However, the strain ID39565 (ST221) was susceptible to penicillin (MIC ≤ 0.06 μg/ml) in the current study. The three serotype 24 strains were susceptible to ceftriaxone, cefepime, levofloxacin, and chloramphenicol. Resistance to tetracycline, erythromycin, azithromycin, and clindamycin was detected for all strains. Worldwide antimicrobial resistance data available for *S. suis* indicate that *S. suis* samples recovered from both humans and pigs have high resistance to tetracycline and moderate to high resistance to macrolides, such as erythromycin (Hoa et al., 2011; Chen et al., 2013b; Hernandez-Garcia et al., 2017; Yongkiettrakul et al., 2019; Ichikawa et al., 2020; Matajira et al., 2020; Dechêne-Tempier et al., 2021).

**FIGURE 2:**
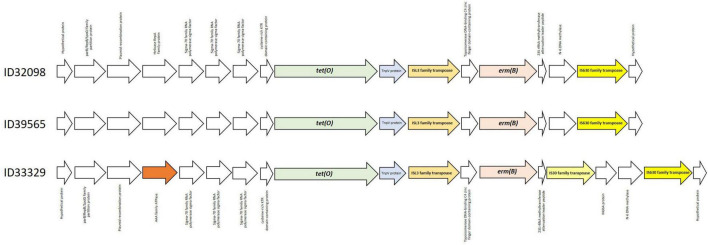
Genetic organization of *tet(O)* and *erm(B)* genes in all three *Streptococcus suis* serotype 24 strains.

ResFinder 4.1 identified *tet(O)* and *erm(B)* which confer to resistance on tetracycline and macrolide-lincosamide-streptrogramin (MLS_*B*_) resistance, respectively, in these three strains. No other antimicrobial resistant genes were detected in these current strains. Several studies have also demonstrated that *tet(O)* and *erm(B)* are commonly found in *S. suis* strains from pigs and humans worldwide (Chen et al., 2013b; Bojarska et al., 2016; Zhang et al., 2020; Dechêne-Tempier et al., 2021; Yongkiettrakul et al., 2021). As shown in Figure 2, the organization of the *tet(O)* and *erm(B)* genes was identical in strains ID32098 and ID39565. ID33329 showed a similar genetic organization to both these strains; however, the genes IS30 family transpoase and MobA protein were inserted between the genes N-6 DNA methylase and 23S rRNA methyltransferase attenuation leader peptide. In addition, the AAA family ATPase gene was observed instead of the helicase RepA family protein gene in the strain ID33329. The *erm(B)* gene was flanked by the ISL3 family transpoase and IS630 family transpoase genes in ID32098 and ID39565; however, the *erm(B)* gene of ID33329 was flanked by the ISL3 family transpoase and IS30 family transpoase genes.

### Analysis of Mobile Genetic Elements and Defense Systems

PlasmidFinder revealed no REP type plasmid in the strains. However, PLACNETw identified MOB_*P*_ and MOB_*V*_ in all three genomes of the serotype 24 strains based on mobility types according to the amino acid sequences of the relaxase proteins. Most of the integrative and conjugative elements (ICEs) found in *S. suis* genomes encoded a canonical relaxase of the MOB_*P*_ family, in contrast with integrative and mobilizable elements (IMEs). Diversity of canonical relaxase of the MOB_*C*_, MOB_*V*_, or MOB_*Q*_, or non-canonical relaxase (MOB_*T*_) was detected (Libante et al., 2019). All three serotype 24 strains seem to contain ICE with MOB_*P*_ and IME with MOB_*V*_.

All strains contained five insertion sequence (IS) families, namely, ISL3, IS110, IS982, IS4, and IS200/IS605. CRISPRCasFinder showed one CRISPR element with a DR (direct-repeat) consensus sequence length of 24 bp (AGCTTGTAGGGCGTGTTCAATTCC) in the strains ID39565 and ID32098, while strain ID33329 contained 2 CRISPR elements with a DR consensus sequence length of 24 bp (GGAATTGAACACGCCCTACAAGCT) and 48 bp (GGTCACATAGAAATGTGAAA-GTGACCATTTGAAAACC CGAGCTTGAAA), respectively. The CRISPR/Cas system has been reported in several *S. suis* serotypes and clonal complexes (Okura et al., 2017; Zhu et al., 2021). A recent study demonstrated that RM and CRISPR/Cas systems were related to the *S. suis* clade and that CRISPR components were absent in clade 1 *S. suis* strains but present in all clade 2 strains (Zhu et al., 2021). The CRISPR DR consensus sequence length of 36 bp (GTTTTACTGTTACTTAAATCTTGAGAGTACAAAAAC) was present in *S. suis* clade 2, that was different from our strains which contained either 24 or 48 bp (Zhu et al., 2021).

None of the strains contained any RM system based on using the Restriction-Modification finder. Toxin-antitoxin (T-A) and abortive infection (Abi) systems, which defend against invading genetic material through different mechanisms from those of the aforementioned systems by limiting phage spread via altruistic cell suicide, were also detected in the genomes of ID33329 (JM964_03790, JM964_03810, JM964_04400), ID39565(KPA27_05665, KPA27_06250, KPA27_06265), and ID32098 (JSY00_05380, JSY00_05960, JSY00_05975). Both systems have been identified in *S. suis*, with either a T-A or Abi systems coexisting with the RM system (Okura et al., 2017). The diversity of these defense elements described above may be related with phenotypic differences in various clades or lineages of the *S. suis*.

### Virulence-Associated Genes

Twenty-four out of 80 VAG were absent in all three strains consisting of: adhesin P, *epf, hyl, ihk, irr, mrp, nadR, neuB, NisK/R, ofs*, permease *(SSU0835), rgg, revS, salK/R, sao, sly, spyM3_0908, srtBCD, srtF, srtG, SSU05_0473, virA, virB4*, and *virD4* (Supplementary Table 1). The classical VAG pofile (*epf/sly/mrp*) was completely absent in our strains. A recent study suggested that *epf, mrp*, and *sly* are mostly associated with serotype 2 and 14 strains but not in other serotypes (Estrada et al., 2021). That study proposed *ofs* and *srtF* as virulence markers for all serotypes. Unfortunately, none of the three strains in the current study contained these markers, so they may be considered as unknown-pathogenic or commensal pathotypes based on the Estrada scheme (Supplementary Table 1; Estrada et al., 2021). It should be noted that the proposition of such markers was done after the analysis of exclusively North American strains (Estrada et al., 2021). More studies are needed to establish whether both genes are present in European and/or Asian isolates.

Another study described the presence of copper-exporting ATPase 1 and type I restriction-modification system S protein genes as makers for *S. suis* disease-associated strains and a putative sugar ATP-binding cassette transporter gene as a marker for non-disease-associated strains (Wileman et al., 2019). In addition, our strains lacked both disease-associated marker genes suggesting non-disease-association (Supplementary Table 1). However, the strains in the current study were isolated from ill patients, indicating a certain pathogenic potential. Therefore, extensive evaluation of marker genes for pathotyping of *S. suis* serotypes other than serotype 2 should be done.

**FIGURE 3:**
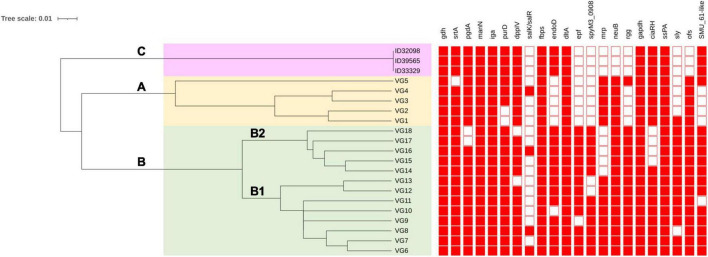
Clustering of *S. suis* serotype 24 strains based on profiles of virulence-associated genes. VG is virulence-associated gene profile. Filled squares refer to presence of virulence-associated genes and blank squares represents absence of virulence-associated genes. Clustering was conducted using the unweighted average linkage (UPGMA) method with DendroUPGMA program. Cluster A, B, and C are shown on the tree.

Dong et al. (2015) have shown 18 profiles of VAG (VG1–VG18) in *S. suis* serotype 2 strains from China based on 22 VAG consisting of: *gdh, srtA, pgdA, manN, iga, purD, DppIV, salK/R, fbps, endoD, dltA, epf, spyM3_0908, mrp, neuB, rgg, gapdh, ciaR/H, SspA, sly, ofs*, and *SMU_61-like*. That study revealed *S. suis* serotype 2 strains could be divided into 2 clusters (A and B) with differences in the distribution of VAG (Dong et al., 2015). As shown in Figure 3, three main clusters (A, B, C) were identified based on the UPGMA tree in the current study. Cluster A consisted of VG1–VG5 with the absence of VAG in the range of 6–8 genes. Cluster B was divided into subclusters B1 and B2. Subcluster B1 contained VG6–VG13 and lacked VAG in the range of 1–3 genes, whereas subcluster B2 consisted of VG14–VG18 and lacked 2–5 VAG. Interestingly, 6 VAGs, *epf, sly, endoD, rgg, SMU_61-like*, and *SpyM3_0908*, were rarely found in cluster A, but in contrast, these genes were highly distributed in cluster B (Dong et al., 2015; Zhu et al., 2021). These six VAGs were found to be characteristic of the virulent group of serotype 2 strains (Dong et al., 2015). Cluster C contained our three strains that lacked 8 VAG of the Dong scheme: *salK/salR, epf, spyM3_0908, mrp, neuB, rgg, sly*, and *ofs*. A recent study showed that *S. suis* serotypes 3 and 7 had VAG profiles classified into cluster I or cluster A in the current study (Zhu et al., 2021). In contrast with our serotype 24 strains, the VAG profile was distinguished into cluster C with the absence of *neuB* and *ofs* that were present in clusters A and B. Overall, clusters C and A lacked some similar VAG components; however, the presence of *endoD* and *SMU_61-like* in cluster C was lacking in cluster A; similarly, *mrp* and *neuB* in cluster A were absent in cluster C.

Variation in the VAG distribution may have been associated with the virulence or pathogenesis of *S. suis*, as diseased-associated *S. suis* strains seem to contain more virulence factors than non-diseased associated strains (Weinert et al., 2015). A zebrafish model demonstrated that cluster B was virulent, whereas cluster A showed relatively low virulence (Dong et al., 2015). Based on these results, the virulence of the strains in cluster C needs to be clarified in the future. However, these three strains were isolated from patients, indicating they have a certain pathogenic potential.

### Genomic Comparison

MLST analysis of our three *S. suis* serotype 24 strains confirmed two ST221 (ID33329 and ID39565) and one ST234 (ID32098) strains that belonged to CC221/234. Analysis of the MCG group showed that all strains were MCG group 7 (subgroup 7–3). This MCG7-3 group contained diverse *S. suis* strains and carried the lowest number of VAG, especially the 3 classical VAGs, *epf*, *sly*, and *mrp* (Chen et al., 2013a; Zheng et al., 2014a). This may indicate that our strains should be considered as intermediate/weakly virulence (I/WV) concordant with a previous study (Chen et al., 2013a). In addition, the CDS2157 gene encoding RNA binding S1, present in all I/WV and virulent (V) strains but not in the epidemic or highly virulent (E/HV) strains described elsewhere, was analyzed in the current study (Zheng et al., 2014a). All three serotype 24 strains contained the CDS2157 gene, suggesting that they may be either I/WV or V group (Supplementary Table 1).

**FIGURE 4:**
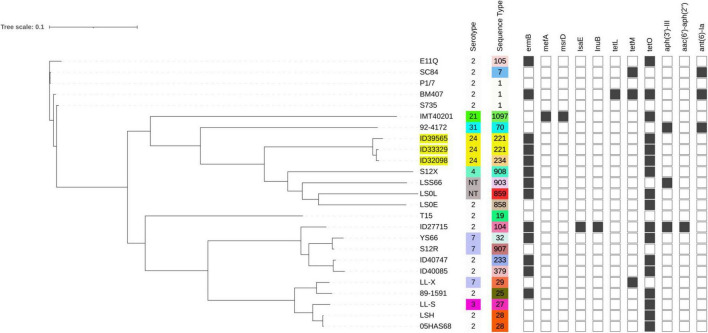
Whole-genome phylogeny analysis of *S. suis* serotype 24 and close relatives generated by CSI Phylogeny and visualized with interactive tree of life tool. The whole genome sequence of *S suis* serotype 24 strains in the current study are highlighted in yellow. Antimicrobial-resistant genes were shown in each strains. Filled squares refer to presence of antimicrobial-resistant genes and blank squares represent absence of antimicrobial-resistant genes.

As shown in Figure 4, the serotype 24 strains were clustered together with the closest relatives of strains S12X, LSS66, LS0L, LS0E, 92–4172, and IMT40201. This cluster contained different serotypes and STs. These closest relative’s strains were from pigs. SNP differences of our three serotype 24 strains compared to the type strain S735 were 13,582, 13,674, and 13,699 SNPs for ID32098, ID33329, and ID39565, respectively. Among the closest relative’s strains, strain LS0E (ST858) was very closely related to our three serotype 24 strains than to others with different SNPs of 10,302, 10,328, and 10,546 for ID33329, ID32098, and ID39565, respectively. The serotype 24 cluster was distinguished from cluster of avirulent (T15 and 05HAS68) or I/WV (89–1591), while the cluster of E/HV (SC84, P1/7) was located far from other clusters. It might be that these serotype 24 strains are in the I/WV group following their position in the tree and the description above. In addition, the clinical data of these three patients indicated that they had predisposal conditions such as alcohol abuse in two patients (ID33329 and ID32098) and liver cirrhosis in the third patient (ID39565) (Kerdsin et al., 2018). These underlying conditions could increase the susceptibility to the infection in these patients.

**FIGURE 5:**
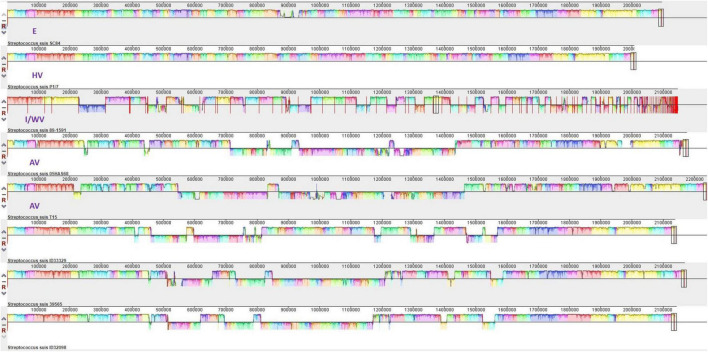
Multigenome comparisons among *S. suis* serotype 24 strains ID33329, ID39565, ID32098, and serotype 2 stain SC84, P1/7, 89–1591, T15, and 05HAS68 performed using the Mauve software. Each color region refers to a locally collinear block (LCB). Colors are arbitrarily assigned to each LCB by the software. Vertical peaks in each LCB denote the variance of conservation. LCBs below the centerline of genomes are in reverse complement orientation.

We compared these three serotype 24 genomes with the representative genomes of E strain SC84 (ST7), HV strain P1/7 (ST1), I/WV strain 89–1,591 (ST25), and avirulent strains T15 (ST19) and 05HAS68 (ST28). As shown in Figure 5 and Table 1, in total, 11 coding sequences or genes were present in only the serotype 24 strains and were absent in the representative strains of E, HV, I/WV, and avirulent. These genes encoded a YSIRK-type signal peptide-containing protein, a KxYKxGKxW signal peptide domain-containing protein, a low temperature requirement protein A, an ABC transporter ATP-binding protein/permease, an aquaporin, helix-turn-helix transcriptional regulator, a Cd(II)/Zn(II)-sensing metallo regulatory transcriptional regulator CadX, a CadD family cadmium resistance transporter, a SpaH/EbpB family LPXTG-anchored major pilin, a class C sortase, and a hypothetical protein. However, we detected unique sequences only in ID32098 and ID39565. PTS sugar/fructose/glucitol transporter and aldose 1-epimerase family protein genes (JSY00_01370-JSY00_01385), and clumping factor (JSY00_10255) were present in only ID32098, whereas the bacteriophage cluster gene (KPA27_02655-KPA27_02925) was detected in ID39565.

**TABLE 1:** Distribution of 11 specific genes identified in *S. suis* serotype 24 strains of the current study and other *S. suis* strains available in the GenBank database using BLASTN search.

No	Locus tag in ID33329	Locus tag in ID32098	Locus tag in ID39565	Gene product	Homology to *S. suis* strain	Serotype	Sequence type	Source	Country	Coverage (%)	Identity (%)
1	JM964_01325	JSY00_01325	KPA27_01335	Aquaporin	1081	31	ND	Pig	China	100	97
					0061	31	ND	Pig	China	100	97
					NJ3	ND	240	Pig	China	100	97
					9401240	9	220	Human	The Netherlands	100	96
					SS389	ND	ND	Pig	China	100	96
2	JM964_01330	JSY00_01330	KPA27_01340	Helix-turn-helix transcriptional regulator	–	–	–	–	–	–	–
3	JM964_01335	JSY00_01335	KPA27_01345	Cd(II)/Zn(II)-sensing metalloregulatory transcriptional regulator CadX	CZ130302	Chz	383	Pig	China	100	99
					9401240	9	220	Human	The Netherlands	100	98
4	JM964_01340	JSY00_01340	KPA27_01350	CadD family cadmium resistance transporter	CZ130302	Chz	383	Pig	China	100	99
					9401240	9	220	Human	The Netherlands	100	98
5	JM964_07190	JSY00_02590	KPA27_02575	YSIRK-type signal peptide-containing protein	WUSS351	ND	ND	Pig	China	100	98
					HN105	5	498	Pig	China	100	96
					HA1003	4	1006	Pig	China	99	97
					9401240	9	220	Human	The Netherlands	99	96
					1081	31	ND	Pig	China	99	96
					0061	31	ND	Pig	China	99	96
					SS389	ND	ND	Pig	China	99	96
					NJ3	ND	240	Pig	China	99	96
6	JM964_07185	JSY00_02595	KPA27_02580	Hypothetical protein	HA1003	4	1006	Pig	China	100	88
					0061	31	ND	Pig	China	83	89
					1081	31	ND	Pig	China	83	89
					HN105	5	498	Pig	China	83	89
7	JM964_07180	JSY00_02600	KPA27_02585	KxYKxGKxW signal peptide domain-containing protein	9401240	9	220	Human	The Netherlands	92	82
					HA1003	4	1006	Pig	China	92	82
					HN105	5	498	Pig	China	92	86
					SS389	ND	ND	Pig	China	92	83
					NJ3	ND	240	Pig	China	92	81
					WUSS351	ND	ND	Pig	China	91	83
8	JM964_06745	JSY00_03035	KPA27_03305	SpaH/EbpB family LPXTG-anchored major pilin	9401240	9	220	Human	The Netherlands	100	99
					1081	31	ND	Pig	China	100	99
					0061	31	ND	Pig	China	100	99
					WUSS351	ND	ND	Pig	China	100	99
					HN105	5	498	Pig	China	100	99
					SS389	ND	ND	Pig	China	100	99
					NJ3	ND	240	Pig	China	100	98
					HA1003	4	1006	Pig	China	100	98
9	JM964_06740	JSY00_03040	KPA27_03310	Class C sortase	NJ3	ND	240	Pig	China	100	99
					HA1003	4	1006	Pig	China	100	99
					SS389	ND	ND	Pig	China	100	98
					HN105	5	498	Pig	China	100	98
					WUSS351	ND	ND	Pig	China	100	98
					1081	31	ND	Pig	China	100	98
					0061	31	ND	Pig	China	100	98
					9401240	9	220	Human	The Netherlands	100	98
1110	JM964_02680	JSY00_07075	KPA27_07305	ABC transporter ATP-binding protein/permease	YSJ17	ND	1071	Pig	China	99	72
					AKJ18	ND	ND	Pig	China	99	72
					AH681	Chz	475	Pig	China	99	72
					1112S	ND	1615	Pig	China	99	71
					CZ130302	Chz	383	Pig	China	99	71
					HN136	Chz	264	Pig	China	99	71
					GZ0565	9	243	Pig	China	98	71
					DN13	9	243	Pig	China	98	71
					DAT300	ND	115	Pig	Japan	98	71
					LS9N	ND	890	ND	United Kingdom	98	71
					FJSM5	ND	1593	Pig	China	97	71
11	JM964_06365	JSY00_03415	KPA27_03685	Low temperature requirement protein A	LS9N	ND	890	ND	United Kingdom	100	95
					YZDH1	ND	ND	Pig	China	100	95
					WUSS351	ND	ND	Pig	China	100	94
					HN105	5	498	Pig	China	100	94
					FJSM5	ND	1593	Pig	China	100	94
					AH681	Chz	475	Pig	China	99	92
					AKJ18	ND	ND	Pig	China	98	95

*ND, No Data.*

Of the 11 specific-genes found in all serotype 24 strains in the current study, three regions were classified, namely, R1 (the aquaporin, transcriptional regulator, CadX, and CadD), R2 (the YSIRK- and KxYKxGKxW-signal peptide domain-containing protein and the hypothetical protein), and R3 (the SpaH/EbpB family LPXTG-anchored major pilin, and the class C sortase) (Table 1 and Supplementary Table 2). BLASTN analysis revealed that R1 (2,055 bp) was highly specific in this sequence to our strains only. The cadmium resistance transporter (*cadD*) and its regulator (*cadX*) of R1 were present in the serotypes 24 strains (100% identity) and *S. suis* strains CZ130302 and 9401240 (isolated from pig in China and human in the Netherlands, respectively), with 99% identity (Table 1). The aquaporin and the transcriptional regulator genes of R1 could be detected in 75–84% of the sequence coverage in *S. suis* strains 1081, 0061, 9401240, NJ3, and the SS389 (Table 1).

LPXTG or related motif gene-coding proteins including a SpaH/EbpB family LPXTG-anchored major pilin, class C sortase, YSIRK- and KxYKxGKxW type signal peptide-containing proteins have still unknown functions, but these proteins have been suggested as being associated with host cell adhesion and invasion (Fischetti et al., 1990; Gagic et al., 2013). BLASTN searching revealed that some *S. suis* strains also contained these protein genes with an identity range of 95–100%, including strains SS389, HN105, WUSS351, HA1003, NJ3, 9401240, 1081, and 0061, as shown in Table 1.

The low temperature requirement protein A has been found to be essential for growth at low temperatures as reported in *Listeria monocytogenes* (Zheng and Kathariou, 1995). Our study showed 100% identities of the genes in *S. suis* strains WUSS351, HN105, LS9N, YZDH1, FJSMS, and AH681 (Table 1). ABC transporter ATP-binding protein/permease plays an important role in the transportation of various substrates, serves as an efflux pump, or is involve in biofilm formation (Chaiden et al., 2021). Another study reported that the presence of ABC transporters in the virulent-to-humans SS2, SS14 strains, and non-virulent-to-humans SS7 strains indicated their possible involvement for the survival and then the pathogenesis of virulent *S. suis* in the host-simulated environment (Chaiden et al., 2021). We detected the ABC transporter ATP-binding protein/permease gene in several *S. suis* strains such as YSJ17, AKJ18, AH681, HN136, LS9N, and CZ130302 (Table 1).

## Conclusion

Genomic exploration of three *S. suis* serotype 24 of CC221/234 revealed 11 specific sequences that were found in the serotype 24 genomes that are different from those of the representative E, HV, I/WV, and avirulent strains. Based on the schematic systems of pathotyping and the VAG profile used in the serotype 2, the three serotype 24 CC221/234 strains may be classified as in the V group or the I/WV group. These strains belong to MCG7-3 and carried *tet*(O) and *ermB* for resistance to tetracycline, macrolide, and lincosamide.

## Data Availability Statement

The original contributions presented in the study are publicly available. This data can be found here: National Center for Biotechnology Information (NCBI) BioProject database under accession number PRJNA691075 (CP068708, CP076517, and CP082778 for the strain no. ID33329, ID39565 and ID32098, respectively).

## Ethics Statement

Ethical review and approval were not required because no human specimens or data were used in the current study.

## Author Contributions

AK: conceptualization and resources. AK, PC, and RH: formal analysis. AK, RH, TW, and PJ: investigation. AK, TW, PJ, and PB: methodology. AK, HZ, and NF: validation. AK, HZ, NF, and MG: writing—original draft, writing—review and editing. All authors contributed to the article and approved the submitted version.

## Conflict of Interest

The authors declare that the research was conducted in the absence of any commercial or financial relationships that could be construed as a potential conflict of interest.

## Publisher’s Note

All claims expressed in this article are solely those of the authors and do not necessarily represent those of their affiliated organizations, or those of the publisher, the editors and the reviewers. Any product that may be evaluated in this article, or claim that may be made by its manufacturer, is not guaranteed or endorsed by the publisher.

## Footnotes

1
https://github.com/rrwick/Porechop

2
https://github.com/wdecoster/NanoPlot

3
https://www.bioinformatics.babraham.ac.uk/projects/fastqc/

4
https://pubmlst.org/organisms/streptococcus-suis

5
http://www.genomicepidemiology.org/services/

6
https://crisprcas.i2bc.paris-saclay.fr/

7
https://blast.ncbi.nlm.nih.gov/Blast.cgi
